# PReS-FINAL-2095: Older age predicts poor response to 6-months methotrexate therapy in a juvenile idiopathic arthritis cohort of patients

**DOI:** 10.1186/1546-0096-11-S2-P107

**Published:** 2013-12-05

**Authors:** R Marques, F Ramos, I Perpétuo, S Fernandes, C Furtado, AF Mourão, F Martins, H Canhão, JE Fonseca, JA Pereira da Silva

**Affiliations:** 1 Rheumatology Research Unit, Instituto de Medicina Molecular - Faculdade de Medicina da Universidade de Lisboa, Lisbon, Portugal; 2 Rheumatology Department, Lisbon Academic Medical Centre, Lisbon, Portugal; 3 Rheumatology, Portuguese Society of Rheumatology, Lisbon, Portugal

## Introduction

The identification of predictive factors of poor response to methotrexate (MTX) in juvenile idiopathic arthritis (JIA) patients could contribute to optimize the treatment strategy, namely by the earlier introduction of biological treatments.

## Objectives

To identify baseline clinical or laboratorial predictive factors of MTX poor response at 6 months in patients with JIA.

## Methods

In a cohort of 141 JIA patients registered in Reuma.pt (the Portuguese Register of Rheumatic Diseases) we selected patients diagnosed after 2000 and that were treated at least for 6 months with MTX (at least 10 mg/m^2^). Univariate and multivariate logistic analyses were used to identify the predictors for non-response to MTX treatment during the first 6 months of treatment. Non-response to MTX was defined according the American College of Rheumatology pediatric 70 (ACR-ped 70) criteria. Concomitant treatment with nsaids and corticosteroids up to 10 mg was allowed for all treatment groups. For this analysis we included: sex, age, age at disease onset, body surface, JIA subtype, disease duration, rheumatoid factor (RF), ANA, HLA-B27, uveitis, age at MTX onset, MTX dosage, disease duration until the start of MTX, baseline physician and patient/parents VAS, baseline active and limited joints and ESR.

## Results

From the 58 patients identified, 23 were excluded (2 started biological therapy before 6 months, 3 were on concomitant DMARD's, 12 had insufficient data, 1 developed criteria for systemic lupus erythematosus, 3 had interrupted MTX and 2 were loss for follow-up). From our population 43% have achieved ACR-ped 70 response and the remaining 57% did not reach this response criterion. The age at MTX onset (p = 0.03; responders 6.25 ± 5, non-responders 9.67 ± 1.53) and body surface (p = 0.045; responders 0.92 ± 0.36; non-responders 1.3 ± 0.26) were predictors of ACR-ped 70 response. Gender, MTX dosage, JIA subtype and the other covariates described above did not interfere on patients' response to MTX. We studied the association of the 2 significant predictors (age at MTX onset and body surface) with response in a multivariate model, but they behave as collinear variables, loosing significance by increasing the standard error. Thus we kept in the model age at MTX onset adjusted to the potential confounders gender, JIA subtype and disease duration until start MTX. Age at MTX onset has remained an independent significant predictor of ACR response (p = 0.037, OR 0.777).

## Conclusion

MTX response was not dependent on subtype, gender and dosage. Older age at MTX onset was correlated with poorer response to a 6-month MTX course, adjusted to gender and subtype.

## Disclosure of interest

None declared.

